# Pyk2 Stabilizes Striatal Medium Spiny Neuron Structure and Striatal-Dependent Action

**DOI:** 10.3390/cells10123442

**Published:** 2021-12-07

**Authors:** Shannon L. Gourley, Kolluru D. Srikanth, Ellen P. Woon, Hava Gil-Henn

**Affiliations:** 1 Yerkes National Primate Research Center, Department of Pediatrics, Emory University School of Medicine, 954 Gatewood Rd. NE, Atlanta, GA 30329, USA; ellen.woon@emory.edu; 2 The Azrieli Faculty of Medicine, Bar-Ilan University, Safed 1311502, Israel; dutt415@gmail.com

**Keywords:** Pyk2, FAK, caudate putamen, contingency, learning, memory, reward

## Abstract

In day-to-day life, we often choose between pursuing familiar behaviors that have been rewarded in the past or adjusting behaviors when new strategies might be more fruitful. The dorsomedial striatum (DMS) is indispensable for flexibly arbitrating between old and new behavioral strategies. The way in which DMS neurons host stable connections necessary for sustained flexibility is still being defined. An entry point to addressing this question may be the structural scaffolds on DMS neurons that house synaptic connections. We find that the non-receptor tyrosine kinase Proline-rich tyrosine kinase 2 (Pyk2) stabilizes both dendrites and spines on striatal medium spiny neurons, such that Pyk2 loss causes dendrite arbor and spine loss. Viral-mediated Pyk2 silencing in the DMS obstructs the ability of mice to arbitrate between rewarded and non-rewarded behaviors. Meanwhile, the overexpression of Pyk2 or the closely related focal adhesion kinase (FAK) enhances this ability. Finally, experiments using combinatorial viral vector strategies suggest that flexible, Pyk2-dependent action involves inputs from the medial prefrontal cortex (mPFC), but not the ventrolateral orbitofrontal cortex (OFC). Thus, Pyk2 stabilizes the striatal medium spiny neuron structure, likely providing substrates for inputs, and supports the capacity of mice to arbitrate between novel and familiar behaviors, including via interactions with the medial-prefrontal cortex.

## 1. Introduction

The dorsomedial striatum (DMS) is a striatal compartment that is indispensable for flexible behavior, including favoring behaviors that are likely to be reinforced with desirable outcomes over other, less fruitful behaviors. Damage to the DMS causes rats to pursue familiar behaviors even when they cease to be reinforced [1,2,3], and instrumental conditioning—performing a behavior for reward—triggers immediate early gene expression and transcriptional activity in the DMS [4,5,6]. Similarly, motor task learning recruits neural ensembles in the DMS that decline in activity with task proficiency [7]. Behavioral plasticity based on goal features and reward likelihood requires inputs from the medial prefrontal cortex (mPFC) and certain subregions of the orbitofrontal cortex (OFC) to the DMS [8,9,10]. The way in which striatal neurons host stable connections that are essential for flexible action is still being elucidated. This question is important because alterations or failures in goal-seeking behaviors, or tendencies towards irrational decision making, are features of multiple neuropsychiatric illnesses [11]. An entry point to addressing this question may be to develop a better understanding of the structural scaffolds within DMS neurons that house stable synaptic connections.

Proline-rich tyrosine kinase 2 (Pyk2) is a nonreceptor tyrosine kinase highly expressed in the brain, where it is enriched in asymmetric (presumably excitatory) synapses, relative to symmetric (presumably inhibitory) synapses [12]. With regard to Pyk2, it is activated by Ca^2+^, integrin receptors, and growth factors, triggering dimer assembly and autophosphorylation, and providing a binding site for Src kinase (reviewed in [13]). Src then phosphorylates additional sites on Pyk2, which allows for the full activation of Pyk2 and its binding of downstream cytoskeletal regulatory proteins. Thus, Pyk2 links synaptic plasticity with the regulation of the actin cytoskeleton, the structural lattice that controls the shape and motility of dendritic arbors and spines. Furthermore, postsynaptic proteins postsynaptic density 95 (PSD-95), SAP102, and SAPAP3—for instance, recruiting PSD-95 to synapses and Pyk2 directly interact [12].

The majority of investigations into Pyk2 brain function have focused on the hippocampus, however Pyk2 is also abundant in the striatum [14], where the vast majority (Σ95%) of cells are inhibitory medium spiny neurons (MSNs). Whether Pyk2 impacts MSN shape and complexity (which directly relates to the capacity of neurons to house synapses) is unclear. We report here that Pyk2 is necessary for dendrite complexity and spine abundance on striatal MSNs, such that Pyk2 loss in knockout mice impoverishes the neuron structure. Next, we selectively reduced *Ptk2b,* which encodes Pyk2, in the DMS in order the isolate Pyk2 function in this brain region. *Ptk2b*-deficient mice were unable to arbitrate between rewarded and nonrewarded behaviors, while *Ptk2b* overexpression enhanced that ability. Thus, Pyk2 appears necessary for DMS function. Finally, experiments using combinatorial viral vector strategies suggest that Pyk2 acts by supporting mPFC-to-DMS, but not necessarily OFC-to-DMS, interactions. 

## 2. Methods

### 2.1. Subjects 

In this study, *Ptk2b* ^−/−^ mice were generated as previously described [15]; control mice were wild-type (WT) littermates. Mice for viral vector/behavioral experiments were females bred in-house on a mixed strain background (C57BL/6J;129X1/SvJ). In one instance, viral vectors were infused into *Thy1*-YFP-expressing mice (H line from [16]), in order to illustrate their viral vector spread on a coronal brain section in which landmarks were also highly visible. Wild type and *Thy1*-YFP mice were originally sourced from Jackson Labs and bred in-house. Mice were maintained on a 12 h light cycle (0800 on), experimentally naïve, and were provided food and water ad libitum unless otherwise indicated. Mice were aged 31–60 days at the start of the experiments. 

### 2.2. Golgi-Cox Staining 

WT and *Ptk2b* ^−/−^ mice were deeply anaesthetized and perfused transcardially with phosphate-buffered saline (pH 7.4). Brains were dissected, briefly washed with PBS to remove traces of blood, and placed in impregnation solution. Brains were coronally sectioned into 150 μm sections using a vibrating microtome (Campden Instruments, Manchester, UK) and collected in a mounting buffer. Sections were then mounted on 1% gelatin coated cover glass slides and stained using the superGolgi Kit (Bioenno Lifesciences, Santa Ana, CA, USA) following manufacturer’s instructions. Stained slides were mounted using Ecomount-K (Kaltek). 

### 2.3. Neuron Imaging 

Stack images of neurons and spines from whole-mount sections of dorsomedial and dorsolateral striatum regions were acquired using a Zeiss Apotome microscope with an ORCA-Flash 4.0 V3 camera. To assess the dendritic morphology of striatal MSNs, low magnification (10×/0.3, 20×/0.8, and 40×/0.75) images were acquired as Z-stack with a 0.5 μm interval. Dendritic spines were imaged from tertiary dendrites of MSNs using high magnification imaging (100×/1.4 oil objective, digital zoom 3, Z-stack with 0.255 μm interval). Each dendrite was considered as an independent sample, and 30 μm sections of dendrite were used. Sholl arborization analysis, dendritic length, and spine number and length were compared using the automated filament re-drawing application in Imaris 8.2.1 (Bitplane). The number of dendrites and mice that contributed to each analysis are reported in the figure captions. 

### 2.4. Plasmids

With regard to plasmids, *Ptk2* and *Ptk2b* cDNAs were isolated from mouse embryonic fibroblast cDNA library using PCR with primers containing FLAG tag and sub-cloned into pCSC-SP-PW-CMV-IRES/GFP vector. For shRNA-mediated knockdown, the following sequence was cloned into pLL3.7 lentiviral plasmid: *Ptk2b*-KD, 5′-TACAGTTCCATGATAATCC-3′.

### 2.5. Cell Culture, Transfection, and Viral Vector preparation

The HEK293T cells were cultured in DMEM/10% FBS. Recombinant lentiviruses were produced by co-transfecting cells with lentiviral plasmids containing *Ptk2*, *Ptk2b,* or shRNA sequences along with pDML, pRSV-VSVG, and pCMV-VSVG packaging plasmids using the PEI transfection method. After 24 and 48 h, following transfection, cell supernatent was collected and concentrated using ultracentrifugation (20,000 rpm, 15 °C, 150 min). The virus pellet was resuspended in Hanks balanced salt solution, aliquoted, and frozen at −80 °C.

### 2.6. Western Blot Analysis of Cell Culture and Brain Samples

Anti-Pyk2 was obtained from Cell Signaling Technology (3292); anti-FAK (clone 77; 610088) was obtained from BD Biosciences. Additional anti-FLAG (clone M2; F3165) and an anti-β actin (clone AC-15; A5441) antibody were both obtained from Sigma-Aldrich (St. Louis, MO, USA). Secondary antibodies (goat anti mouse 680LT and goat anti rabbit 800CW) were obtained from Li-COR Biosciences.

Mice were rapidly decapitated, and brains were rapidly frozen at −80 °C for later dissection. Alternatively, transfected HEK293T or snap-frozen lysates were washed in ice-cold PBS and lysed in Triton lysis buffer (1% Triton, 10% glycerol, 120 mM NaCl, 25 mM HEPES, 1 mM EDTA, 0.75 mM MgCl_2,_ 2 mM NaF, 1 mM Sodium orthovanadate, and protease inhibitors). The total protein concentration was determined using a DC protein assay (Bio-Rad Laboratories, Hercules, CA, USA), and equal amounts were loaded on SDS-PAGE, transferred to a nitrocellulose membrane, and blocked in an Odyssey blocking buffer. Membranes were incubated with primary and secondary antibodies and imaged using the Odyssey CLx imaging system (LI-COR Biosciences, Lincoln, NE, USA). The samples were quantified using an Odyssey CLx imaging system (LI-COR Biosciences), and the values were normalized to the loading control and presented as a fold change from the control mean.

### 2.7. Viral Vector Delivery 

The following viral vectors were used in this study: (1) lentiviruses expressing sh-*Ptk2b* or a scrambled control construct and a YFP tag; (2) lentiviruses expressing *Ptk2b* or *Ptk2* or a scrambled control construct and a GFP tag; (3) AAV5-CaMKIIa-hM_4_D(Gi)-mCherry (Gi-coupled DREADDs; see [17] for general review of DREADDs) or AAV5-CaMKIIa-mCherry; and (4) AAV8-CaMKIIa-HA-rM_3_D(Gs)-IRES-mCherry (Gs-coupled DREADDs) or AAV8-CaMKIIa-GFP. In vivo, lentiviruses robustly transfect neurons, although moderate glial transfection would also be anticipated [18]. Lentiviruses were created in house, as described above, and AAVs were sourced from the UNC Viral Vector Core and ADDGene. The same DREADDs-expressing viral vectors were used and further validated in recent prior reports [19,20]. 

Mice were anesthetized with ketamine/dexdomitor and placed in a digitized stereotaxic frame (Stoelting), heads were shaved, scalps incised, skin retracted, and heads leveled. Burr holes were drilled. For DMS infusions, viral vectors were delivered over 10 min in a volume of 1.5 µL at AP + 0.5, DV − 3.0, ML ± 1.7. For mPFC infusions, viral vectors were delivered over 5 min in a volume of 0.5 µL at AP + 2.0, DV − 2.8, ML ± 0.1. For OFC infusions and viral vectors were infused over 5 min in a volume of 0.5 µL at AP + 2.6, DV− 2.8, ML ± 1.2. Needles were left in place for ≥5 additional minutes after infusion and before withdrawal and suturing. After surgery, mice were left undisturbed for Σ1 month, after which either behavioral testing or euthanasia was performed. 

### 2.8. Behavioral Testing 

#### 2.8.1. Food-Reinforced Response Training 

Mice were food-restricted to ~90% of their free-feeding body weight. Mice were trained to nose poke for food reinforcers (20 mg, grain-based Bio-Serv Precision Pellets) in Med-Associates operant conditioning chambers equipped with 2 nose poke recesses and a separate food magazine. Responses was reinforced using a fixed ratio 1 (FR1) schedule such that 30 pellets were available for responding on each of 2 distinct nose poke recesses. Sessions ended at 70 min or when 60 pellets were acquired. Mice were trained for at least 7 sessions, or until they acquired all 60 pellets within the 70 min. We confirmed that mice did not have side preferences that could impact later responding. Response acquisition curves represent both responses/minute for the last 7 training sessions. 

In one experiment, mice were then shifted to a random interval (RI) 30 s schedule of reinforcement for 5 sessions, then an RI60-s schedule for 4 sessions. Both nose poke responses were reinforced, and sessions were again ended when mice acquired 60 pellets, or at 70 min. RI schedules induce responding that is habitual, lacking flexibility and sensitivity to reward availability. 

#### 2.8.2. Test for Behavioral Response Flexibility 

A test of response flexibility was used, as in our prior reports (e.g., [21]), and similar to that of [22] as follows: In a 25 min “contingent” session, one nose poke aperture was occluded, and responses on the other aperture was reinforced using an FR1 schedule of reinforcement, as during training. In the 25 min “noncontingent” session, the opposite aperture was occluded, and pellets were delivered into the magazine at a rate matched to each animal’s reinforcement rate from the previous session. Thus, this response becomes significantly less predictive of reinforcement than the other measured response. This 2-session procedure was conducted twice, with conditions held constant (meaning, one response remained reinforced throughout). The “contingent” vs. “noncontingent” sessions, and which contingency was violated, were counter balanced across the mice. 

The following day, both apertures were made available during a 15 min probe test conducted in extinction. A preferential engagement of the response that is likely to be reinforced reflects response plasticity. Meanwhile, engaging both familiar responses equivalently led to no change from training conditions. In experiments in which mice were subjected to repeated testing, the contingent vs. noncontingent aperture conditions were reversed each time, necessitating an update of the responses, and the probe test was shortened to 10 min to mitigate response extinction with repeated testing.

### 2.9. Clozapine N-Oxide (CNO) Administration and Experimental Design

In experiments using DREADDs, Clozapine N-Oxide (CNO; 1 mg/kg in a volume of 1 mL/100 g, i.p., Sigma-Aldrich) was dissolved in a 2% dimethyl sulfoxide (DMSO, Sigma-Aldrich) solution in 0.9% sterile saline, prepared on the day of injection. The timing of injection was determined based on the existing literature regarding when the mPFC and OFC are thought to contribute to response plasticity. In the case of mPFC experiments, injections were delivered 30 min prior to the sessions when a response was no longer reinforced (based on [23]). In experiments focused on the OFC, injections were delivered immediately following the same sessions, given that the OFC is involved in the post-training updating of expectations (based on [24,25,26]).

In the OFC mice, we detected no effects. To further verify this pattern, we conducted a second probe test the day following the first, administering CNO 30 min prior to the test. Next, we reinstated responses with 4 sessions using an RI30 schedule of reinforcement, and delivered CNO 30 min prior to the violation of a familiar contingency; this condition is referred to as “response reversal”, since the previous reinforced response was no longer reinforced. Response preference was tested the following day, drug free. The timing of these injections is provided in a schematic in the associated figure.

Throughout the experiments, all mice, regardless of viral vector, received CNO, equally exposing them to any unintended consequences of the drug (see [27]). This dose of CNO alone was found to have no effects in this study [24]. Relatedly, Barker et al. [28] found that doubling this dose had no effects in a very similar study. 

### 2.10. Locomotor Activity 

Locomotor activity was monitored using customized Med-Associates chambers, equipped with 16 photobeams. Mice were placed in the chambers for 1 h for habituation. Then, mice were injected with saline, and locomotor activity was monitored for 1 h. After this, mice were administered 3 mg/kg D-amphetamine (i.p., volume 1 mL/100 g), and locomotor activity was monitored for 1 h. A relatively low dose was used to allow for the resolution to detect elevated locomotor activity, if it existed. Photobeam breaks resulting from repeated interruption of a single photobeam (potentially reflecting motor stereotypies) have been reported. 

### 2.11. Histological Verification of Viral Vector Placement 

Mice were euthanized by deep anesthesia, then rapid decapitation. Brains were submerged in 4% paraformaldehyde for 48 h, then transferred to 30% *w/v* sucrose, followed by sectioning into 50 µm-thick sections on a microtome, maintained at a temperature of −15 °C. Viral vector infusion sites were verified by imaging GFP, YFP, or mCherry as appropriate.

### 2.12. Statistical Analysis

The Pyk2, PSD-95, and dendritic spine densities and lengths were compared using an unpaired Student’s *t*-test. Sholl intersections, response rates, and locomotor counts were compared by ANOVA with repeated measures when appropriate. Post hoc *t*-tests were applied following interactions. Any positive results of post hoc comparisons (i.e., *p* ≤ 0.05) are indicated graphically. Bonferroni corrections were applied to Sholl analyses. In one instance, planned comparisons between 2 groups were made by *t*-tests (planned comparisons are noted as such in text). Values > 2 standard deviations above the mean were considered to be outliers and were therefore excluded. As such, one mouse in the mPFC group of the final figure, which generated very high response rates throughout, and one mouse from the contralateral group in the final experiment of the manuscript, were excluded. Behavioral experiments were conducted twice, and both cohorts are represented throughout. Data were analyzed by GraphPad Prism 8.0 or SPSS.

## 3. Results

### 3.1. Pyk2 Is Highly Expressed in the Striatum, Where It Controls Dendrite Arborization and Spine Density on Striatal MSNs

**Figure 1:**
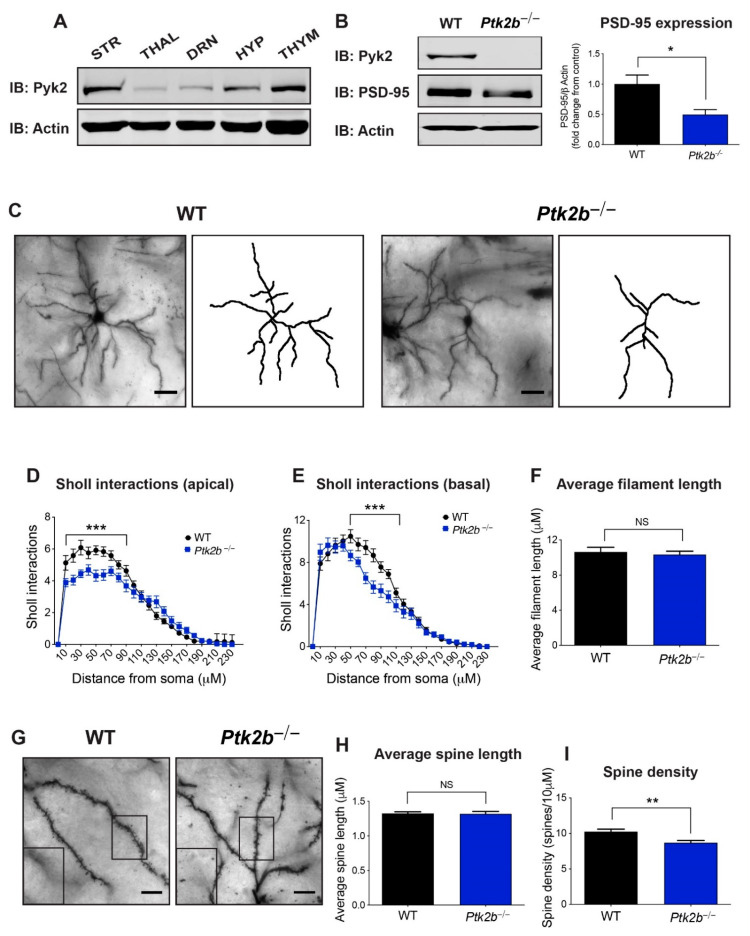
Pyk2 ensures complex dendrite arbors and spines on medium spiny neurons of the DMS. (**A**) Immunoblot analysis of Pyk2 in striatum (STR) compared to other brain regions: thalamus (THAL), dorsal raphe nucleus (DRN), hypothalamus (HYP), and to the thymus, a hematopoietic organ where Pyk2 is highly expressed (THYM). *n* = 3 mice per group. (**B**) Left, representative immunoblots of Pyk2 and PSD-95 levels in WT and *Ptk2b*^−/−^ striatum. Right, quantification of PSD-95 protein levels. *n* = 3 mice per group. (**C**) Representative Golgi-Cox images and tracing of WT (**left**) and *Ptk2b*^−/−^ (**right**) medium spiny dorsomedial striatal neurons. Scale bar, 25 µm. (**D**,**E**) Apical (**D**) and basal (**E**) Sholl interaction analysis of dorsomedial WT and *Ptk2b*^−/−^ striatum neurons. *n* = 28 neurons from 4 mouse brains. (**F**) Average filament length of WT and *Ptk2b*^−/−^ dorsomedial spiny neurons. (**G**) Representative Golgi-Cox images of dendritic spines from WT and *Ptk2b*^−/−^ medium spiny neurons. Scale bar, 10 µm. (**H**,**I**) Quantification of dendritic spine length (**H**) and density (**I**) on medium spiny neurons from WT and *Ptk2b*^−/−^ dorsomedial striatum. *n* = 18 dendrites from 4 mouse brains, * *p* < 0.05, ** *p* ≤ 0.01, *** *p* ≤ 0.001. NS, non-significant. Error bars represent SEM.

A Western blot analysis was used to evaluate the relative expression levels of Pyk2 in the striatum vs. other regions of the brain. As demonstrated in Figure 1A, and in agreement with previous studies [29,30,31], Pyk2 is highly expressed in the striatum relative to thalamus, dorsal raphe nucleus, or hypothalamus, and similar in expression levels to the thymus, a hematopoietic organ in which Pyk2 is highly expressed.

Next, we measured PSD-95, a post-synaptic marker, in the striatum of *Ptk2b*^−/−^ mice, revealing lower levels (*t*_4_ = 3.319, *p* = 0.04) (Figure 1B), as also occurs in the *Ptk2b*^−/−^ hippocampus [12]. Next, we imaged Golgi-Cox-labeled MSNs from WT and *Ptk2b*^−/−^ DMS (Figure 1C). A Sholl analysis revealed less dendritic branching on apical arbors, with primary effects occurring proximal to the soma (distance x genotype interaction *F*_(23,1152)_ = 3.70, *p* < 0.0001; the main effect of distance from the soma *F*_(23,1152)_ = 106.90, *p* < 0.0001; main effect of genotype *F*_(1,1152)_ = 20.31, *p* < 0.0001) (Figure 1D). The same pattern was observed for the basal arbors ((distance x genotype interaction *F*_(23,1248)_ = 3.19, *p* < 0.0001; main effect of distance from the soma *F*_(23,1248)_ = 147.80, *p* < 0.0001; main effect of genotype *F*_(1,1248)_ = 23.37, *p* < 0.0001) (Figure 1E)), though no changes were observed in the average filament length (*t*_47_ = 0.46, *p* = 0.65) (Figure 1F). Similarly, we found no changes in average spine length (*t*_32_ = 0.10, *p* = 0.92) (Figure 1G,H). Spine density was, however, lower in the *Ptk2b*^−/−^ DMS (*t*_32_ = 3.25, *p* = 0.003) (Figure 1I).

**Figure 2:**
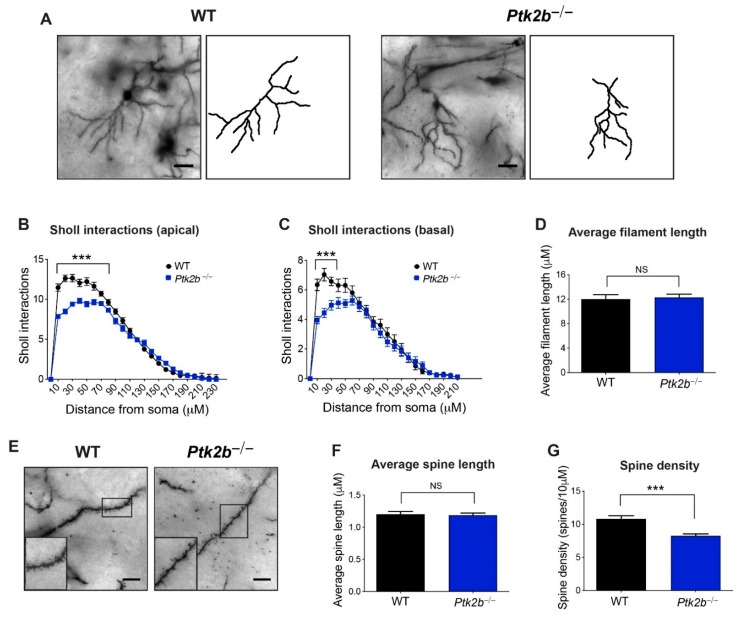
Pyk2 ensures complex dendrite arbors and spines on medium spiny neurons of the dorsolateral striatum. (**A**) Representative Golgi-Cox images and tracing of WT (**left**) and *Ptk2b*^−/−^ (**right**) medium spiny dorsolateral striatal neurons. Scale bar, 25 µm. (**B**,**C**) Apical (**B**) and basal (**C**) Sholl interaction analysis of dorsolateral WT and *Ptk2b*^−/−^ striatum neurons. *n* = 28 neurons from 4 mouse brains. (**D**) Average filament length analysis of WT and *Ptk2b*^−/−^ dorsolateral spiny neurons. (**E**) Representative Golgi-Cox images of dendritic spines from WT and *Ptk2b*^−/−^ medium spiny neurons of the dorsolateral striatum. Scale bar, 5 µm. (**F**,**G**) Quantification of dendritic spine length (**F**) and density (**G**) on medium spiny neurons from WT and *Ptk2b*^−/−^ dorsolateral striatum. *n* = 18 dendrites from 4 mouse brains, *** *p* ≤ 0.001. NS, non-significant. Error bars represent SEM.

Similar phenotypes were observed in the dorsolateral striatum (Figure 2A), including losses in the apical dendrite arborization, proximal to the soma (distance x genotype interaction *F*_(23,1152)_ = 11.55, *p* < 0.0001; main effect of distance from the soma *F*_(23,1152)_ = 415.70, *p* < 0.0001; main effect of genotype *F*_(1,1152)_ = 90.66, *p* < 0.0001) (Figure 2B). Basal arbors were also found to be impoverished (distance x genotype interaction *F*_(21,1078)_ = 3.53, *p* < 0.0001; main effect of distance from the soma *F*_(21,1078)_ = 110.40, *p* < 0.0001; main effect of genotype *F*_(1,1078)_ = 30.31, *p* < 00001) (Figure 2C). The overall filament length did not differ between groups (*t*_38_ = 0.33, *p* = 0.74) (Figure 2D), nor did dendritic spine length (*t*_32_ = 0.27, *p* = 0.79) (Figure 2E,F). Nevertheless, dendritic spines were found to have again been lost (*t*_32_ = 4.72, *p* < 0.0001) (Figure 2G). Thus, it was found that Pyk2 is essential for dendritic complexity and spine abundance on striatal MSNs.

### 3.2. Pyk2 Supports Behavioral Flexibility

**Figure 3:**
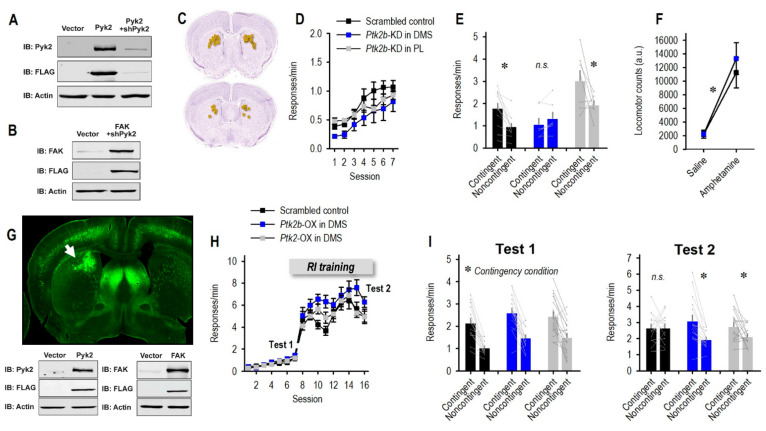
Striatal Pyk2 is necessary for sustained behavioral flexibility. (**A**) Immunoblot analysis of *Ptk2b* knockdown in HEK293T cells. Cells were transfected with a control lentiviral vector, Pyk2-FLAG expressing lentiviral vector, or Pyk2-FLAG expressing vector, together with Pyk2 shRNA lentiviral vector, to determine Pyk2 knockdown efficiency. (**B**) Cells were transfected with a control vector or with a FAK-FLAG expressing vector, together with Pyk2 shRNA, to determine the knockdown specificity of Pyk2 shRNA. As illustrated, the Pyk2 shRNA sequence is specific to Pyk2 and does not deplete the closely related FAK. (**C**) Composite of viral vector infusion sites in the DMS in this report. Each square represents an individual infusion. (**D**) Mice acquired nose poke responses on 2 ports; responses on both ports are collapsed for simplicity. (**E**) When the association between one response and its outcome was violated via noncontingent pellet delivery, control mice and mice with Pyk2 silenced in the prefrontal cortex (PL subregion) inhibited the response. Meanwhile, silencing Pyk2 in the DMS obstructed response plasticity (*n* = 13 combined control group, *n* = 7 DMS, *n* = 7 PL). (**F**) Mice with *Ptk2b* knockdown in the DMS displayed typical locomotor activity, including when treated with amphetamine. (**G**) **Top**: A unilateral viral vector infusion in the DMS is shown in a coronal section from a mouse expressing *Thy1*-driven YFP, to visualize anatomical landmarks. **Bottom**: Immunoblot analysis of Pyk2 and FAK overexpression in HEK293T cells. Cells were transfected with the viral vector control, Pyk2 overexpressing lentivirus, or FAK overexpressing lentivirus. (**H**) Mice were trained to nose poke for food reinforcers, and tests for behavioral flexibility are indicated, as are training sessions using random interval (RI) schedules of reinforcement. (**I**) For the first test, all mice inhibited a behavior when it was unlikely to be reinforced (noncontingent condition). With further training, control mice developed habit-like behavior—indicated by equivalent responses on both apertures, while mice with Pyk2 and FAK overexpression retained behavioral flexibility. Thus, Pyk2 and FAK overexpression improved response flexibility. *n* = 15 control, *n* = 16 Pyk2, *n* = 17 FAK, * *p* < 0.05. KD, knockdown. NS, non-significant. OX, over-expression. PL, prelimbic. Error bars represent SEM. Line connecting bars represent individual mice.

We then aimed to understand the Pyk2 function in the striatum. Given that Pyk2 is expressed throughout multiple brain regions, the most definitive strategy was to selectively reduce Pyk2 in the striatum, leaving it intact in other brain regions. To achieve this objective, we specifically designed shRNAs to reduce the *Ptk2b* in transfected cells. To first assess the efficiency and specificity of sh-*Ptk2b*, HEK293T cells were transiently co-transfected with a functional Pyk2-FLAG, together with sh-*Ptk2b,* and the knockdown efficiency was determined by immunoblotting (Figure 3A). To further confirm the specificity of sh-*Ptk2b*, it was co-transfected with a construct expressing the closely related focal adhesion kinase (FAK). As demonstrated in Figure 3B, sh-*Ptk2b* did not reduce the levels of co-transfected FAK, indicating the high specificity of the *Ptk2b* knockdown sequence.

The striatum is composed of multiple compartments with different functions. A primary function of the DMS is to integrate the necessary inputs for organisms to flexibly select actions based on the likelihood that they will be rewarded. To determine whether Pyk2 is necessary for this process, we used viral vectors to selectively reduce Pyk2 in the DMS (Figure 3C,G). Control mice received either a scrambled control viral vector or sh-*Ptk2b* in the mPFC (primarily, prelimbic cortex) for comparison to a region with lower endogenous Pyk2 levels. mPFC histology is summarized in the figure provided.

Mice were trained to nose poke on two ports for two food reinforcers. Throughout the experiment, no side biases or pellet preferences were detected, thus responses on both apertures have been collapsed for simplicity. The silencing of Pyk2 had no effect on response training (main effect of session *F*_(6,144)_ = 14.22, *p* < 0.001; no effect of group *F*_(2,24)_ = 1.48, *p* = 0.25; no interaction *F* < 1) (Figure 3D). We then violated the predictive relationship between one response and the reward. In reaction, control mice and mice with Pyk2 silencing in the mPFC inhibited the response that was unlikely to be reinforced. In contrast, sh-*Ptk2b* in the DMS occluded the ability of mice to update response strategies (group x contingency condition interaction *F*_(2,24)_ = 5.30, *p* = 0.012) (Figure 3E), such that they responded equivalently on both ports. The main effects of contingency condition and group were also detected (*F*_(1,24)_ = 10.41, *p* = 0.004; *F*_(2,24)_ = 7.17, *p* = 0.001). Thus, Pyk2 in the DMS is necessary for the generation of flexible responses—that is, favoring one familiar behavior over another when adaptive.

Another function commonly ascribed to the dorsal striatum is the execution of motor stereotypies, repetitive behaviors that occur apparently without purpose. We monitored striatal *Ptk2b*-deficient mice in locomotor monitoring chambers. We identified no effects of knockdown, including when mice were primed with amphetamine, which can elicit motor activity ((main effect of amphetamine *F*_(1,11)_ = 44.9, *p* < 0.001; no effect of group or interactions *F*s < 1) (Figure 3F)). This pattern suggests that dorsomedial striatal Pyk2 does not impact the basic motor function, consistent with prior investigations using *Ptk2b*^−/−^ mice and mice with reduced amounts of Pyk2 in the DMS [32]. Importantly, these patterns indicate that the instrumental response patterns described above cannot be definitely attributed to changes in locomotor activity.

### 3.3. Enrichment of Striatal Pyk2 or FAK Improves Action Flexibility

We next aimed to test whether Pyk2 overexpression could enhance behavioral flexibility. We used RI schedules of reinforcement that biased the response strategies towards habitual behaviors, which are by definition insensitive to reward likelihood. Either Pyk2 or the closely related FAK was overexpressed in the DMS (Figure 3G), and mice subsequently acquired the nose-poke responses. Response rates increased across time (*F*_(15,675)_ = 150.54, *p* < 0.001) (Figure 3H). The trending main effects of group and group x session interaction likely reflect slightly higher response rates in the Pyk2 group during RI training (*F*_(2,45)_ = 3.16, *p* = 0.052; *F*_(30,675)_ = 2.25, *p* = 0.12).

We first confirmed that mice were behaviorally sensitive to reward likelihood following a brief period of training, before the RI schedules were used. The point was to confirm that overexpression did not unexpectedly interfere with response flexibility, and indeed, all groups inhibited a behavior that was unlikely to be reinforced (main effect *F*_(1,45)_ = 86.41, *p* < 0.001; no interaction *F* < 1) (Figure 3I, Test 1). Next, mice were shifted to an RI schedule of reinforcement and trained for several more days (Figure 3H). At this point, following RI training, control mice did not modify their response strategies when rewards were withheld—serving as evidence that they had formed habitual routines, as expected. Meanwhile, the overexpression of Pyk2 or the closely related FAK enhanced behavioral sensitivity to reward probability, such that these mice inhibited a behavior that was unlikely to be reinforced (group x contingency condition interaction *F*_(2,45)_ = 4.59, *p* = 0.02; main effect of contingency condition *F*_(1,45)_ = 15.35, *p* < 0.001; no main effect of group *F* < 1) (Figure 3I, Test 2). Overall, Pyk2 appears to exert bidirectional control over response flexibility, —the response was found to be impaired with reduced levels of Pyk2, whereas it was improved for higher levels.

### 3.4. mPFC Stimulation Reinstates Flexible Behavior following Striatal Pyk2 Loss

**Figure 4:**
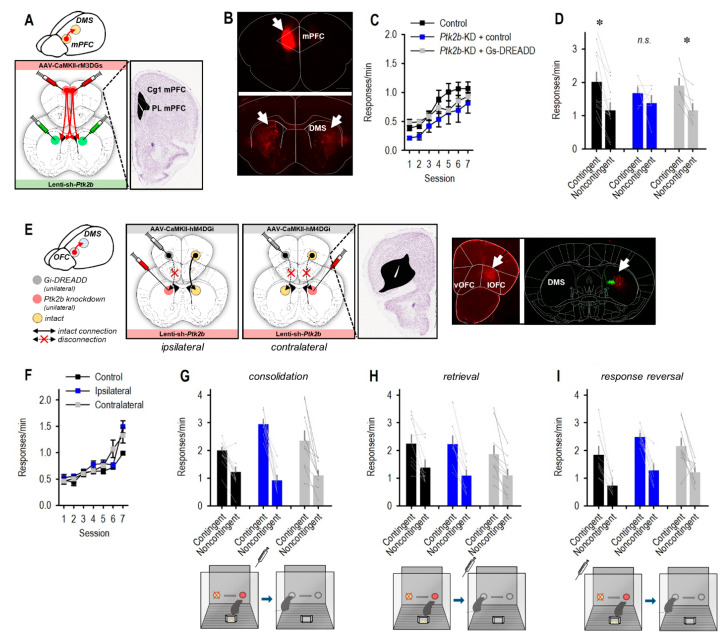
Coordination with the mPFC, but not OFC, in striatal Pyk2-dependent flexible behavior. (**A**) Pyk2 was silenced in the DMS as in Figure 3. Simultaneously, DREADDs-expressing viral vectors were placed in the mPFC, primarily prelimbic cortex (PL). Large and small viral vector spread is represented. (**B**) This experimental design was motivated by evidence that the mPFC (within the white lines) sends monosynaptic projections to the ipsilateral and contralateral DMS. Viral vector placement in the ipsilateral mPFC at the top, with bilateral terminals at the bottom (arrows). (**C**) Response acquisition in the absence of CNO did not differ between groups. Mice with control viral vectors in the DMS and either a control viral vector or a Gs-DREADD-expressing viral vector in the mPFC (and a control viral vector in the DMS) did not differ and were combined into a single control group. (**D**) The DREADD ligand CNO was delivered prior to the period when pellets were delivered noncontingently. Mice were tested in the probe test, drug free, the following day. Control mice inhibited a behavior that was not reinforced (noncontingent condition), while *Ptk2b* knockdown obstructed response flexibility, as in Figure 3. Simultaneous stimulation of the mPFC via Gs-coupled DREADDs restored response flexibility (*n* = 11 combined control group, *n* = 6 DREADD + *Ptk2b* knockdown, *n* = 6 control viral vector + *Ptk2b* knockdown). (**E**) The OFC and DMS are connected via unidirectional projections that are largely ipsilateral, lending the next experiment to a disconnection experimental design in which Pyk2 is silenced in the DMS of one hemisphere and Gi-DREADDs are placed in the OFC of one hemisphere. The contralateral condition tests whether Pyk2-dependent action selection requires inputs from the OFC. Representative OFC infusion sites and spread are adjacent. At **right**: Unilateral infusion of an mCherry-expressing viral vector in the OFC results in detectable terminals in the ipsilateral DMS (red), corresponding with the site of *Ptk2b* knockdown (green), but not the contralateral DMS. (**F**) Mice were trained to nose poke for food in the absence of CNO. (**G**) CNO was delivered at multiple time points, represented by the cartoons below each graph, ultimately revealing no effects on memory consolidation, (**H**) memory retrieval, or (**I**) a response reversal. *n* = 9 control, *n* = 8 ipsilateral, *n* = 10 contralateral, * *p* < 0.05. Cg1, cingulate cortex, area 1. DMS, dorsomedial striatum. KD, knockdown. lOFC, lateral orbitofrontal cortex. mPFC, medial prefrontal cortex. NS, non-significant. PL, prelimbic cortex. vOFC, ventral orbitofrontal cortex. Error bars represent SEM. Line connecting bars represent individual mice.

mPFC-to-DMS and OFC-to-DMS connections are required for certain aspects of action flexibility. We aimed to test whether plasticity in the mPFC or OFC was necessary for Pyk2-dependent action selection. We used two strategies, which were determined based on the anatomical organization of cortico-striatal connections, arising from the mPFC vs. OFC. The mPFC sends dense monosynaptic projections to the unilateral and contralateral DMS—meaning that some projections are contained within one hemisphere, while roughly a quarter of projections comprise of crossing fibers [8] (Figure 4A,B). Thus, we first infused control or sh-*Ptk2b*-expressing viral vectors bilaterally into the DMS. In half of the mice, we also infused “excitatory” Gs-coupled DREADDs or a control viral vector in the mPFC (Figure 4A). The two groups of mice that expressed control viral vectors in the DMS (±Gs-DREADDs in the mPFC) did not differ and were combined.

Mice were trained to nose poke for food in the absence of the DREADDs ligand CNO. They acquired the reinforced responses without group differences ((main effect of session *F*_(6,120)_ = 34.14, *p* < 0.001; no main effect of group *F* < 1; no interaction *F*_(2,20)_ = 1.3, *p* = 0.23) (Figure 4C)). The DREADDs ligand CNO was delivered immediately before the contingency violation. Response strategies were measured the next day when mice were drug-free. We detected a main effect of the contingency condition and no interaction or group effects (*F*_(1,20)_ = 22.46, *p* < 0.001; *F*_(2,20)_ = 1.6, *p* = 0.23; *F* < 1, respectively), given that most mice preferred the response that was likely to be reinforced (Figure 4D). Nevertheless, planned comparisons indicated that Pyk2 silencing in the absence of DREADDs in the mPFC blocked response plasticity, as shown in Figure 3 (*t*_5_ = 1.02, *p* = 0.36). Meanwhile, simultaneous stimulation of Gs-DREADDs in the mPFC safeguarded response preference (*t*_5_ = 3.74, *p* = 0.01) (Figure 4D). Control mice also preferred the highly reinforced response, as expected (control *t*_10_ = 4.61, *p* < 0.001) (Figure 4D). Thus, Pyk2-dependent flexibility appears to be sensitive to mPFC inputs, such that mPFC stimulation can compensate for Pyk2 loss.

OFC-to-DMS projections are also implicated in goal seeking [9,10], and these projections are organized in a largely ipsilateral fashion [26], making them particularly amenable to “disconnection” experimental designs (Figure 4E). In this case, control or sh-*Ptk2b*-expressing viral vectors were infused into the DMS of one hemisphere, while control or inhibitory Gi-coupled DREADDs were placed in the OFC of one hemisphere. When the infusions were contralateral, the Pyk2-deficient DMS received input from a healthy OFC, while OFC input to the healthy DMS was disrupted due to Gi-DREADDs (Figure 4E). If this combination of viral vectors disrupted response strategies, the interpretation would be that Pyk2-dependent action selection requires inputs from the OFC. Control mice received the same sh-*Ptk2b*- and DREADDs-expressing viral vectors, but they were contained within a single hemisphere, leaving the other intact. A final control group received control viral vectors in either ipsilateral or contralateral hemispheres.

All groups acquired the nose poke responses (main effect of session (*F*_(6,144)_ = 53.65, *p* < 0.001), but no group differences were detected (no main effect of group (*F*_(2,24)_ = 3.01, *p* = 0.07; no interaction *F*_(2,24)_ = 3.14, *p* = 0.06) (Figure 4F). CNO was delivered immediately following the contingency violation to potentially disrupt stable memory formation. This specific timing was selected because bilateral OFC silencing at the same time obstructs response updating in the same task [24,25,26]. Upon the probe test, however, all groups were sensitive to reward contingency, with no effects of “disconnection” (group *F* < 1), and only a robust main effect of the contingency condition (*F*_(1,24)_ = 94.5, *p* < 0.001) (Figure 4G). This outcome suggests that Pyk2-dependent action is not obviously dependent on OFC input.

For thoroughness, we repeated the procedure, except providing CNO upon the probe test, when mice must retrieve memories regarding outcome expectancies. Again, we detected no effect of group (*F* < 1), and only a main effect of contingency condition (*F*_(1,24)_ = 45.3, *p* < 0.001) (Figure 4H). Finally, we reinstated responding and lastly, we retested sensitivity to contingency violation, delivering CNO ahead of a violation of the previously intact contingency (termed “reversal”). Still, all groups were able to inhibit a response that was unlikely to be reinforced (main effect of contingency condition *F*_(1,23)_ = 56.85, *p* < 0.001) (Figure 4I). A main effect of the group was also detected, but post hoc comparisons were found to be non-significant (*F*_(2,23)_ = 4.14, *p* = 0.03). Thus, the effects of Pyk2 in supporting response flexibility do not obviously involve the OFC.

## 4. Discussion

Goal-directed behavior refers to the selection of actions based on the anticipation that they will result in desirable outcomes; these behaviors are flexible and modulable, based on expectations [33]. For instance, we no longer use a faulty vending machine that fails to deliver our preferred snack, or we might avoid behaviors that are dangerous—such as driving without a seatbelt. Stressors and addictive drugs cause failures in goal-directed behavior in rodents and humans alike [34,35,36]. Cortico–striatal projections are necessary for goal seeking, leading us to hypothesize that cytoskeletal regulatory elements—meaning, the molecules that control the presence and stability of dendritic arbors and spines that receive incoming projections—would control behavioral flexibility (a necessary aspect of goal seeking) in mice.

Here, we investigated Pyk2, an FAK family nonreceptor tyrosine kinase that is closely related to FAK. Pyk2 is highly expressed in excitatory post-synaptic densities in neurons [12,37]. Unlike several cytoskeletal regulatory factors that decline in expression during early postnatal life, Pyk2 levels are found to be robust in mature animals [14], potentially positioning it to control dendritic spine structure and synaptic plasticity across the lifespan. In this study, the loss of Pyk2 impoverished striatal MSNs, the primary cell type in the striatum, causing dendritic arbor and spine loss. Thus, Pyk2 stabilizes neuron structure in the striatum. As would be expected by the loss of dendritic spines, PSD-95, a postsynaptic scaffolding protein commonly used as a synaptic marker, was also lost, suggesting that Pyk2 supports synaptic presence in the striatum, as in the hippocampus [12]. Interestingly, in a prior report, PSD-95 was found to be unaffected in striatal extractions from *Ptk2b*^−/−^ mice [32]. In this report, de Pins et al. used 4-month-old mice. As reported by these authors in previous publications, and also discussed by our group in two recent publications [13,38], a compensatory effect of the closely related FAK may begin in older age and take over the regulation of neuronal morphology.

A synapse-protective role for Pyk2 has been documented in multiple investigations. For instance, dendrites on pyramidal CA1 hippocampal neurons of *Ptk2b^−/−^* mice suffer a loss of dendritic spines and PSD-95 clusters; moreover, Pyk2 restoration in the hippocampi of Huntington’s disease model mice, which exhibit low Pyk2, partially rescues this same phenotype [12]. Importantly, though, other studies using Alzheimer’s disease (AD) model mice suggest a detrimental role for Pyk2 on synapse maintenance. An overexpression of Pyk2 in the hippocampi of AD model mice leads to synapse loss via the inhibitory interaction of Pyk2 with Graf1, which leads to increased RhoA activity and consequent actomyosin contractility and dendritic spine retraction [39]. Furthermore, deletion of Pyk2 in these mice rescues synaptic loss and learning and memory deficits [40]. Overall, the precise impact of Pyk2 on neuronal structure and stability likely depends on age, brain region, and disease state.

We next aimed to understand the Pyk2 function in the striatum. Given that Pyk2 is expressed throughout multiple brain regions, the most effective strategy was to selectively reduce Pyk2 in the striatum, leaving it intact in other brain regions. As discussed in the Introduction, the DMS subregion controls behavioral flexibility. Pyk2 loss here eliminated the ability of mice to modify familiar behaviors based on their likely consequences. More specifically, mice were unable to favor behaviors that were highly reinforced, relative to those that were not. The same patterns occurred following the site-selective inhibition of the dendritic spine stabilizing factors Abl2 kinase and tropomyosin receptor kinase B [41,42], leading us to posit that the dendritic spine-destabilizing effects of Pyk2 silencing likely account for poor flexibility in knockdown mice.

Next, to determine whether Pyk2 overexpression in the DMS could enrich flexible behavior, we utilized schedules of reinforcement that generate strong or weak associations between actions and their outcomes. A fixed ratio (FR) schedule means that a response will be regularly reinforced according to a fixed probability. For instance, an FR1 schedule (as used here) means that every response will be reinforced. In contrast, in interval schedules, an interval of time during which reinforcement is not available is inserted between reinforcer deliveries and thus, resources are not found to be freely available. Ratio schedules form strong associations between responses and their outcomes, while interval schedules weaken the control of action–outcome associations over behavior [33]. We trained mice to nose poke according to RI schedules of reinforcement, including mice with Pyk2 overexpressed in the DMS. The enrichment of Pyk2 caused mice to retain behavioral sensitivity to the links between actions and outcomes, even while control mice lost such sensitivity and instead utilized habit-like response strategies.

Pyk2 and FAK comprise the FAK family of non-receptor tyrosine kinases and share 48% amino acid sequence identity and common phosphorylation sites and similar domain structures. Despite these similarities, their expression patterns differ (reviewed in [13]). Firstly, FAK is expressed in most cells, while Pyk2 is more restricted to the central nervous system and hematopoietic cells. Secondly, FAK levels are fount o be high during embryonic development, after which they taper off. Meanwhile, Pyk2 levels are extremely low early in development and then increase markedly as animals mature to adulthood [14]. These expression patterns led us to focus on Pyk2 throughout most of these investigations, particularly when using gene silencing approaches, in which case, loss of Pyk2 represents a significant departure from physiological conditions (while loss of FAK would not, since levels are already low). In our overexpression experiments, however, we included FAK. Similar to Pyk2, an overexpression of FAK in the DMS of adult mice enhanced the capacity of mice to track outcome availability, presumably acting via the stabilization of neurite extensions and synaptic function [43].

The DMS is the site of convergence of cortical projections necessary for different components of flexible behavior. The mPFC is generally thought to encode action–outcome memories necessary for flexible goal seeking (reviewed in [44]). The mPFC sends dense monosynaptic projections to the unilateral and contralateral DMS, and the crossing fibers appear to be most critical for goal-oriented choice [8]. We hypothesized that Pyk2 deficiency could impede the ability of mice to execute choice by destabilizing mPFC-to-DMS connections. In this case, Pyk2 loss might necessitate a stronger mPFC signal for flexible action to occur. Indeed, the chemogenetic stimulation of excitatory mPFC neurons blocked response failures in mice suffering striatal Pyk2 deficiency.

We also tested the whether Pyk2-dependent action requires inputs from the ventrolateral OFC. This cortical region is generally associated with modifying behaviors when familiar rules and contingencies change [45], and there is some evidence that it could also be involved in learning certain reward-related associations ([46]; see for further discussion [47]). Mice in our task must modify their response strategies, shifting from the equivalent execution of two behaviors, to preferring one behavior when they learn that the other is no longer reinforced, so investigating the OFC seemed sensible. Furthermore, OFC-striatum interactions are necessary for response switching based on outcome value [9,10], and the suppression of compulsive-like behavior, which can interfere with goal-directed action [48].

Unlike mPFC-to-DMS projections, OFC-to-DMS projections are overwhelmingly ipsilateral [49], allowing us to use a functional “disconnection” strategy to determine whether striatal Pyk2 sustains functional sensitivity to OFC inputs. We delivered inhibitory Gi-DREADDs to one OFC and silenced Pyk2 in the contralateral DMS. In the presence of the DREADD ligand CNO, healthy OFC projections terminate on Pyk2-deficient DMS neurons in one hemisphere, while the OFC is inactive in the opposite hemisphere. If Pyk2-dependent action requires OFC input, these conditions will block the ability of mice to update response preferences. We administered the DREADD ligand CNO at multiple time points—when new memories were being formed, when they were being stabilized (consolidated), and when they were being retrieved to guide choice. No condition was found to have any effect, suggesting that Pyk2 control over response flexibility does not definitely require OFC inputs.

Notably, the disconnection strategy used here disrupts the response selection in the same task when lesions are placed in the contralateral OFC and dorsal striatum [50]. Furthermore, bilateral lesions and chemogenetic silencing of the OFC—particularly the ventrolateral sub-region targeted in this study—disrupt response strategies in the same task [24,25,26]. Finally, this same batch of viral vector was used to disrupt behavioral plasticity in another investigation in our lab [20], so we believe that the null effects are not simply a failure of experimental design or tools.

What might account for apparently dissociable roles for the mPFC and OFC in influencing Pyk2-dependent action? A potential factor is the distribution of D1R- and D2R-expressing MSNs. These neurons are overwhelmingly segregated and Pyk2 is expressed on both [32,51], but notably, D1R-expressing MSNs receive huge inputs from the mPFC (particularly cingulate subregion), relative to even other cortical regions that also preferentially innervate D1R+ MSNs [52]. Conceivably, Pyk2 stabilizes the dendritic spines on D1R-expressing MSNs that house synapses receiving mPFC inputs, allowing for the integration of new information into goal-seeking strategies. Interestingly, cocaine increases Pyk2 expression and phosphorylation in the ventral striatum, where it potentiates the locomotor response to cocaine [32,53]. Our experiments, selectively over-expressing Pyk2, suggest that these changes in the DMS could motivate goal seeking, including, potentially, for cocaine.

Notably, Pyk2-loss impoverished neurons in both the medial and lateral dorsal striatal compartments. These striatal subregions have distinct functions, with the DMS controlling goal-seeking behavior, and the DLS controlling habitual behavior [25,54]. Psychostimulants cause dendritic spinogenesis in the DLS [55]; whether Pyk2 is involved could be further investigated. Supporting the possibility, stress localizes Pyk2 to amygdalar synapses, contributing to the spinogenic effects of stress in this brain region [56], and cocaine and other stimulants elicit a stress hormone release [57]. Cocaine-induced dendritic spine plasticity in other brain regions has been causally linked with habit-like behavior [23,58] and also drug-seeking behavior [59,60,61]—calling for further investigations into striatal Pyk2 function.

## Data Availability

Data supporting the reported results can be requested from the corresponding authors.
